# Robotic-assisted retroperitoneal lymph node dissection for testicular cancer: benefit and limitation

**DOI:** 10.1097/JS9.0000000000001174

**Published:** 2024-02-12

**Authors:** Cheuk-Kwan Sun, I-Wen Chen, I-Ting Tsai, Kuo-Chuan Hung

**Affiliations:** a Department of Emergency Medicine, E-Da Dachang Hospital, I-Shou University; b School of Medicine, College of Medicine, I-Shou University, Kaohsiung City; c Department of Anesthesiology, Chi Mei Medical Center, Liouying, Tainan City; d Department of Emergency Medicine, E-Da Hospital, I-Shou University, Kaohsiung City, Taiwan

*Dear Editor*,

The article published in the *International Journal of Surgery* by Ge *et al*.^1^, titled ‘The role of robotic retroperitoneal lymph node dissection in testicular cancer: a systematic review and meta-analysis,’ caught our attention for its thorough evaluation of robot-assisted versus open and laparoscopic retroperitoneal lymph node dissection (RPLND) in the treatment of testicular cancer. The authors reported that, compared to open RPLND, robot-assisted RPLND was associated with significantly shorter hospital stays, less blood loss, and fewer overall complications without differences in operative time, nodal yields, or recurrence rates^1^. When compared to laparoscopic RPLND, robotic-assisted RPLND had higher nodal yields but no other outcome differences^1^. Given the excellent prognosis of testicular cancer when managed correctly and the increasing adoption of minimally invasive surgeries for complex RPLND procedures, the study by Ge *et al*.^1^ is highly relevant and timely.

That meta-analysis^1^ found that patients undergoing robotic-assisted RPLND had a significantly shorter length of hospital stay compared to those receiving open RPLND, with a mean difference of −1.21 days (95% CI −1.66, −0.76). This indicates that, on average, the use of the robotic surgical approach reduced hospitalization by more than 1 day. The shorter length of stay with robot-assisted RPLND can likely be attributed to the minimally invasive nature of the technique compared to open surgery. Shorter hospital stays are an important healthcare quality metric and a priority for enhanced recovery protocols. Reduced hospital stay also enhances patient satisfaction and lowers healthcare expenses. The availability of robot-assisted RPLND could meaningfully impact the length of stay metrics for institutions that perform a high volume of RPLND for testicular cancer. Despite this encouraging finding, there was significant heterogeneity among the included studies (*I*^2^=78.9%)^1^. This indicates variability in the effect sizes, somewhat conflicting results, and potential influencing factors that were not accounted for. Additionally, because all the included studies in that meta-analysis were small retrospective analyses rather than prospective randomized trials, additional analysis may be required.

Calculating a prediction interval, which was not conducted in the original meta-analysis^1^, in addition to the confidence interval could be useful to further examine the robustness of their findings. The confidence interval estimates the mean effect size, whereas the prediction interval assesses the effect in a new study, accounting for between-study heterogeneity^2–4^. A wide prediction interval reflects greater uncertainty regarding the replicability of the effect size that adversely affects the decision to implement an intervention in practice. Accordingly, we recalculated the prediction interval using the raw data from the initial meta-analysis^1^ using the Comprehensive Meta-Analysis software (Version 4, Biostat, Englewood, NJ, USA). Our analysis revealed a 95% prediction interval ranging from −3.22 to 0.79 (Fig. 1), suggesting the need for more evidence to corroborate the original meta-analysis findings. In summary, the meta-analysis by Ge *et al*.^1^ demonstrated an association of robot-assisted RPLND with shorter hospital stays, the variability in this outcome across different studies indicates a need for more comprehensive randomized trials to solidify this finding.

**Figure 1:**
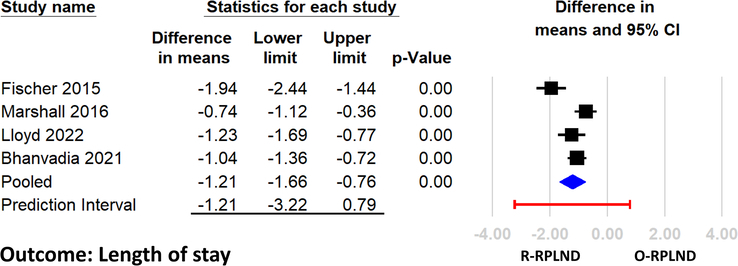
Forest plot comparing the length of stay between robotic-assisted retroperitoneal lymph node dissection (R-RPLND) and open RPLND (O-RPLND) across multiple studies. The plot depicts the difference in means and 95% confidence intervals (CIs) for each study (represented by squares and extending lines) and the pooled effect (blue diamond). The prediction interval (the horizontal red line) spans from −3.22 to 0.79, indicating the expected range of differences in length of stay if the intervention was applied in different settings. This wider interval suggests considerable variation across different contexts despite the average beneficial effect, highlighting the need for cautious interpretation before generalizing these findings.

## Ethical approval

Not applicable.

## Consent

Not applicable.

## Sources of funding

No external funding was received for this study.

## Author contribution

K.-C.H. and C.-K.S.: wrote the main manuscript text; I-T.T. and I-W.C.: prepared Figure 1. All authors read and approved the final version of the manuscript.

## Conflicts of interest disclosure

The authors declare no conflicts of interest.

## Research registration unique identifying number (UIN)

Not applicable.

## Guarantor

Kuo-Chuan Hung.

## Data availability statement

The datasets used and/or analyzed in the current study are available from the corresponding author upon reasonable request.

## Provenance and peer review

This paper was not invited.
